# Evaluating the Association Between Acne Vulgaris and Diet: An Exploratory Study on Patient Beliefs and Perceptions

**DOI:** 10.1111/jocd.70285

**Published:** 2025-07-08

**Authors:** Masomeh Salemi, Sahar Dadkhahfar, Zohreh Tehranchinia, Zahra Razzaghi, Fariba Ghalamkarpour

**Affiliations:** ^1^ Skin Research Center Shahid Beheshti University of Medical Sciences Tehran Iran; ^2^ Laser Application in Medical Sciences Research Center Shahid Beheshti University of Medical Sciences Tehran Iran

**Keywords:** acne vulgaris, belief, diet, perception

## Abstract

**Background:**

Although there is content diversity, there are several data about the impact of diet on acne. However, studies about patients' beliefs about the effects of diet on acne are limited. So, in this study, we aimed to assess the perceptions and beliefs of patients about the association between acne and diet.

**Methods:**

It was a cross‐sectional study that was conducted on patients with a diagnosis of acne vulgaris in the dermatology clinic of Shohada Tajrish and Loghman Hakim Hospital (Tehran, Iran) from December 2022 to December 2023. A questionnaire was given to each patient, and basic data and beliefs of patients about the association between acne and diet were asked. All questionnaires were recorded in SPSS v.26 software and analyzed. A significant level was considered less than 0.05.

**Results:**

In this study, 793 patients participated in the study. 328 patients (41.36%) were 12–19 years old, and 482 of all patients (60.78%) were male. Most of the patients believed that sweets and chocolates and fatty and fried foods had negative impacts on acne and that fruits and vegetables, vitamins, and supplements (zinc, omega‐3, selenium, and green tea) improved the disease condition. Acne severity and skin type had significant relationships with the beliefs of patients about the effects of diet on acne (*p*‐values < 0.05).

**Conclusion:**

Although a considerable number of patients with acne believe that diet impacts acne, the patients should be educated about dietary habits that improve or aggravate the disease.

## 1 Introduction

Acne vulgaris is a complex inflammatory skin disorder, affecting 85% of patients aged 12–24 years [1]. Psychological stress, lifestyle, occupational exposure, medications, environmental pollutants, climate change, and diet can influence acne and its severity [2].

It is reported that the etiology of acne vulgaris entails the interplay of various factors, such as the activation of sebaceous glands due to androgens, immunological responses, and dysbiosis of the pilosebaceous microbiome. Furthermore, factors such as genetics and foods may also affect the disease's development and progression [3, 4]. So, dietary restrictions have been recommended as a part of acne treatment, emphasizing the limitation of carbohydrates, fats, and high‐fat dairy products [5].

The association between diet and acne has been a prominent subject in acne epidemiology research. Currently, numerous studies have established that a high‐sugar diet and dairy products are risk factors for acne. Elevated sugar consumption (≥ 100 g/d), regular consumption (≥ 7 times per week) of soft beverages (including carbonated sodas, sweetened teas, and fruit‐flavored drinks), and daily intake of dark chocolate were strongly correlated with acne [6, 7, 8, 9, 10]. However, data about the effects of different foods and supplements are not similar in different studies, and there are some diversities about the effects of foods on acne [11, 12, 13].

Recent studies demonstrated the relationship between diet and acne, but the patients' beliefs and perceptions about the effects of diet on acne were not assessed enough, and data about it is limited [14]. For example, Smollich et al. determined patient perceptions about acne and diet. They showed that 75% of interviewees assert a correlation between acne and food. Patients pursue supporting information primarily from Instagram (63%), internet forums (54%), and textbooks (46%). Participants perceive their nutritional interventions as ineffective and believe an information gap exists [15]. Therefore, in this study, we assessed patients' beliefs and perceptions about the impact of diet on acne.

## 2 Methods

This cross‐sectional study was performed on patients who were diagnosed with acne vulgaris at the dermatology clinic of Shohada Tajrish and Loghman Hakim Hospital (Tehran, Iran) from December 2022 to December 2023.

The inclusion criteria were a definite diagnosis of acne vulgaris or having a history of acne involvement, referring to the hospitals from December 2022 to December 2023, and an age between 12 and 45 years. The exclusion criteria were not having consent to participate in the study, lack of education to read or write the questionnaire, and failure to cooperate with the physical examination.

A questionnaire was designed for patients, and its content is seen in the supporting Information table (Data S1). The questionnaire consisted of three sections: demographic information, skin characteristics, and beliefs about the influence of dietary habits on acne. The first two sections were completed by a dermatologist. Patients' beliefs and perceptions were assessed by having the patients fill out the third part.

The severity of acne was assessed as mild, moderate, and severe [16], by the dermatologist, and skin type was categorized as normal, dry, oily, or mixed [17].

Two expert groups, four nutrition professors, and four dermatologists evaluated the questionnaire to establish its content validity, assessing its relevance, necessity, and quality. We found the Content Validity Ratio (CVR) and the Content Validity Index (CVI) for questions 10 through 34. The CVR values were above the critical level of 0.75, which means that most of the items had strong content validity. We excluded item 34 due to its insufficient validity. Cronbach's alpha measured the internal consistency, which was exceptionally high at 1.02.

### 2.1 Ethical Considerations

This study was approved by the ethics committee of Shahid Beheshti Medical University (IR.SBMU.MSP.REC.1403.090).

### 2.2 Statistical Analysis

We recorded and analyzed the data using SPSS software version 26. Descriptive statistics (mean, standard deviation, and frequency (percentage)) and inferential statistics (chi‐square tests) were used. A significance level of 0.05 was considered.

## 3 Results

### 3.1 Basic Data of Participants

The study included a total of 793 participants, with 482 (60.78%) males and 311 (39.22%) females. Table 1 shows basic data of all patients.

**TABLE 1:** Basic data of patients.

Variable	Value	Frequency (%)
Gender	Male	482 (60.78%)
	Female	311 (39.22%)
Age	12–19 years old	328 (41.36%)
	20–29 years old	191 (24.09%)
	30–39 years old	247 (31.15%)
	40–45 years old	27 (3.40%)
BMI	< 18.5	123 (15.51%)
	18.5–24.9	362 (45.65%)
	25–29.9	253 (31.90%)
	≥ 30	55 (6.94%)
Education	< High school	4 (0.50%)
	High school	345 (43.51%)
	University	444 (55.99%)

### 3.2 Clinical Data of Participants

Table 2 demonstrates the data about current and previous clinical data of the patients based on the history and physical examination.

**TABLE 2:** Current and previous clinical data of the patients.

Variable	Value	Frequency (%)
History of acne	Had before	424 (53.47%)
	Have now	131 (16.52%)
	Had before and have now	238 (30.01%)
Severity of acne	Severe	31 (3.91%)
	Moderate	384 (48.42%)
	Mild	378 (47.67%)
Skin type	Normal	159 (20.05%)
	Dry	108 (13.62%)
	Oily	269 (33.92%)
	Mixed	257 (32.41%)
Family history of acne	Yes	353 (44.51%)
	No	440 (55.49%)

### 3.3 Patients' Beliefs About Diet and Acne

We assessed patients' beliefs about the effect of diet on acne based on the questionnaire, and the related data are seen in Table 3. Out of all patients, 657 (82.85%) believed that diet can improve acne. 241 (30.39%) and 166 (20.93%) patients “significantly” and “absolutely” agreed with the belief that “dietary habits affect acne.”

**TABLE 3:** Patients' beliefs about the impact of diet on acne.

Variable	Value	Frequency (%)
Can dietary habits affect acne?	Never	23 (2.90%)
	Little	60 (7.57%)
	Sometimes	303 (38.21%)
	Significant	241 (30.39%)
	Absolutely	166 (20.93%)
Does dietary change improve acne?	Yes	657 (82.85%)
	No	136 (17.15%)
Spices	Makes it worse	272 (34.30%)
	Makes it better	21 (2.65%)
	No effect	204 (25.73%)
	No comment	296 (37.33%)
Sweets and chocolate	Makes it worse	601 (75.79%)
	No effect	77 (9.71%)
	No comment	115 (14.50%)
Snacks (chips and puffs)	Makes it worse	457 (57.63%)
	No effect	163 (20.55%)
	No comment	173 (21.82%)
Nuts	Makes it worse	388 (48.93%)
	Makes it better	36 (4.54%)
	No effect	181 (22.82%)
	No comment	188 (23.71%)
Sunflower seeds	Makes it worse	466 (58.76%)
	Makes it better	14 (1.77%)
	No effect	118 (14.88%)
		195 (24.59%)
Non‐alcoholic drinks	Makes it worse	189 (23.83%)
	Makes it better	6 (0.76%)
	No effect	257 (32.41%)
	No comment	341 (43.00%)
Milk	Makes it worse	67 (8.45%)
	Makes it better	80 (10.09%)
	No effect	288 (36.32%)
	No comment	358 (45.15%)
Caffeine	Makes it worse	102 (12.86%)
	Makes it better	24 (3.03%)
	No effect	350 (44.14%)
	No comment	317 (39.97%)
Spicy foods	Makes it worse	265 (33.42%)
	Makes it better	8 (1.01%)
	No effect	261 (32.91%)
	No comment	259 (32.66%)
Fatty and fried foods	Makes it worse	484 (61.03%)
	No effect	151 (19.04%)
	No comment	158 (19.92%)
Eggs and meat	Makes it worse	96 (12.11%)
	Makes it better	56 (7.06%)
	No effect	346 (43.63%)
	No comment	295 (37.20%)
Butter and cream	Makes it worse	376 (47.41%)
	Makes it better	18 (2.27%)
	No effect	164 (20.68%)
	No comment	235 (29.63%)
Fruits and vegetables	Makes it worse	11 (1.39%)
	Makes it better	454 (57.25%)
	No effect	175 (22.07%)
	No comment	153 (19.29%)
High‐salt foods	Makes it worse	119 (15.01%)
	Makes it better	6 (0.76%)
	No effect	331 (41.74%)
	No comment	337 (42.50%)
Verjuice and lemon juice	Makes it worse	18 (2.27%)
	Makes it better	181 (22.82%)
	No effect	297 (37.45%)
	No comment	297 (37.45%)
Fast food	Makes it worse	488 (61.54%)
	Makes it better	4 (0.50%)
	No effect	127 (16.02%)
	No comment	174 (21.94%)
Dates	Makes it worse	241 (30.39%)
	Makes it better	72 (9.08%)
	No effect	256 (32.28%)
	No comment	224 (28.25%)
Vitamin A	Makes it worse	20 (2.52%)
	Makes it better	337 (42.50%)
	No effect	132 (16.65%)
	No comment	304 (38.34%)
Vitamin B	Makes it worse	35 (4.41%)
	Makes it better	279 (35.18%)
	No effect	141 (17.78%)
	No comment	338 (42.62%)
Antioxidants (vitamins E, selenium and green tea)	Makes it worse	11 (1.39%)
	Makes it better	432 (54.48%)
	No effect	94 (11.85%)
	No comment	256 (32.28%)
Omega‐3 (fish and fatty fish)	Makes it worse	90 (11.35%)
	Makes it better	300 (37.83%)
	No effect	132 (16.65%)
	No comment	271 (34.17%)
Zinc	Makes it worse	36 (4.54%)
	Makes it better	399 (50.32%)
	No effect	94 (11.85%)
	No comment	264 (33.29%)

### 3.4 Relationship Between Historical and Clinical Data With the Questionnaire Data

We assessed relationships between basic and clinical data of patients with the results of questionnaires. Due to the expanse of the analyses, we describe the results of the analyses. We found there were no relationships between age, gender, BMI, and education level with total perceptions and beliefs of patients about the impact of diet on acne (*p* value: 0.62, *p* value: 0.57, *p* value: 0.92, and *p* value: 0.90, respectively), but in subgroups, some relationships were found that are described below.

Acne severity and skin type had statistically significant relationships with the total perceptions and beliefs of patients about the effects of diet on acne. The findings indicated that 82 individuals (10.34%) with mild acne, 80 individuals (10.09%) with moderate acne, and 4 individuals (0.5%) with severe acne perceived that dietary habits could “absolutely” impact the exacerbation or improvement of acne. These results about “very high” impact on the exacerbation or improvement of acne were observed in 114 (14.38%), 119 (15.01%), and 8 (1.01%) of cases with mild to severe cases, respectively (*p* value: 0.00). Regarding skin type, 83 (10.47%) of participants with dry skin, 211 (26.61%) of participants with mixed skin, 144 (18.16%) of participants with normal skin, and 219 (27.62%) of participants with oily skin believed that changing their eating habits “absolutely” impacted the exacerbation or improvement of acne (*p* value: 0.01).

About subgroups of the questionnaire, we found that males were more likely than females to believe that snacks, spicy foods, fatty and fried foods, vitamin B, verjuice and lemon juice, and fatty food worsen acne (*p* values < 0.05). Patients with high BMI had higher beliefs about the effects of fatty and fried foods on the worsening of acne (*p* value: 0.00). The education level had statistically significant relationships with the perception and belief of patients about the effects of food consumption on acne in the subgroups of spicy foods, fatty and fried foods, fruits and vegetables, and butter and cream. Patients with higher education levels had more belief in the effects of these foods on the worsening of acne (all *p* values < 0.05).

## 4 Discussion

In this study, which was performed to evaluate the perception and belief of patients with acne about the relationship between acne and diet, it was observed that patients believed that some foods and supplements have a significant impact on the disease, especially sweets and chocolates, which were most commonly believed to exacerbate acne, while fruits and vegetables were recognized to be beneficial for improving acne. Moreover, most participants believed that snacks and fried/fatty foods, spices, and nuts exacerbated acne. We also found that age, gender, BMI, and education level had no significant relationships with the beliefs of patients, generally. In this respect, acne severity and skin type had significant relationships with the beliefs of patients about the effects of diet on acne.

Karciauskiene et al. evaluated perceptions and beliefs of patients about acne in Lithuania. They evaluated the beliefs of 1277 students aged 7 to 19 years, and 51.4% responded to their questionnaire. They found that patients with moderate or severe acne involvement were more aware of the disease. Based on the results of the questionnaire, patients believed that hormones, poor hygiene, and diet were the main causes of acne exacerbation [18]. In the current study, we evaluated the belief of patients about the relationship between acne and diet in detail, and it was one of the advantages of our study. Similar to Karciauskiene et al. we found that higher acne severity had a significant relationship with the belief of patients about the impact of diet on acne. The participants' views on a potential connection between acne and nutrition are notably correlated with their motivation to seek information from supplementary sources [19], and it appears that patients with more severe forms of acne are more likely to receive data about dietary information. These searches increase the knowledge of patients about the impact of diet on acne and affect patients' beliefs in this regard.

Burris et al. assessed patients' beliefs about the relationship between dietary factors and acne severity. They found that participants with moderate to severe acne reported higher consumption of added sugar, total sugar, daily milk servings, saturated fat, and trans‐fatty acids, as well as fewer daily servings of fish compared to those with minimal or mild acne. Among all participants, 58.1% said that food exacerbates or affects acne [6]. These findings were confirmed in the current study. We found that 82.85% of our cases believed that dietary habits can affect or improve acne conditions, and they reported that dietary habits were associated with acne severity.

Markovic et al. evaluated patients' beliefs about acne. They evaluated 452 schoolchildren aged 14 to 18 years, and 55.2% were girls. There were significant differences between boys and girls in terms of beliefs. Boys believed that dairy foods and smoking worsen acne, but girls believed that fatty foods and sweets have a negative impact on acne [20]. In the current study, we found that men believed that spicy foods, snacks, fatty and fried foods, vitamin B, verjuice and lemon juice, and fast food had a negative impact on acne significantly more than females. These findings were different from the study of Markovic et al. This difference may come from the difference between the study populations of the studies. Our study was conducted on 12‐ to 45‐year‐old patients, but Markovic et al.'s study was done on 14‐ to 18‐year‐old patients. This difference can demonstrate that patients' beliefs change over time.

Al‐Natour assessed the beliefs of 329 Saudi adolescent men aged 13 to 22 years. The results of his study showed that 71.5% of patients believed that diet affects acne, and 21% believed that drinking mineral water improves acne severity [21]. Our study was done on both genders, and we found that 20.93% of our patients believed that diet “absolutely” affects acne and 30.39% believed that diet “significantly” affects acne. Also, 38.21% of our patients believed that diet “sometimes” can affect acne. The findings of these two studies about the belief of the effect of diet on acne in patients were relatively similar. The findings suggest that dermatologists should conduct thorough consultations regarding the influence of diet on acne to address patients' medical and nutritional needs. Nutrition counseling for acne patients must address the existing lack of knowledge and mitigate the risk of imposing potentially harmful dietary restrictions.

According to our participants' perceptions, sweets and chocolates, snacks, and sunflower seeds had the most significant impact on exacerbating acne. Furthermore, according to the participants' opinions, antioxidants, fruits and vegetables, and zinc had the most significant impact on improving acne. In this regard, several studies suggest that chocolate consumption is associated with an increase in acne among men with a history of common acne [8, 22]. The study by Vongraviopap et al. found a significant correlation between white chocolate consumption and acne exacerbation, particularly in adolescents and young adults [8]. However, it should be noted that the results of studies are not similar. For example, Suppiah et al. reported that consumption of carbonated drinks, chips, potatoes, sweets, nuts, ice cream, and yogurt had no association with acne, and only chocolate and/or milk consumption was associated with the disease [11]. So, it seems that further studies are still needed in this respect.

Nguyen et al. evaluated 49 patients, and it was found that 92% of patients believed that diet affects acne and that fatty and fried foods, chocolates, dairy products, and soda drinks worsen acne [23]. We evaluated 793, and 2.9% of our patients believed that diet has no effect on acne. Based on our findings, our patients believed that sweets and chocolates, snacks, fried and fatty foods, spices, and nuts exacerbate acne. Although our study had a larger sample size, the findings of these two studies were relatively similar. Patients harbor misunderstandings regarding acne vulgaris. Accessible and precise community‐oriented educational activities can enhance awareness [24].

Consumption of sunflower seeds appears to increase the severity of common acne. In a randomized controlled trial, participants who consumed daily sunflower seeds showed a significant increase in their Acne Severity Index (ASI) compared to the control group [25]. These findings are consistent with the belief of the majority of our participants.

Antioxidants, whether topical or systemic, appear to be effective against acne by reducing oxidative stress and inflammation [26, 27, 28]. Researchers recommend vitamins and supplements like selenium as effective agents for acne treatment [29]. In our study, we observed that less than 60% of our patients believed that vitamins, antioxidants, and supplements are beneficial for the improvement of acne. So, these findings demonstrate the need to increase patients' knowledge about the effects of vitamins, antioxidants, and supplements on acne.

We found that patients with higher BMI believed that fatty and fried foods worsen acne, but Raditra et al. found that there was no significant relationship between BMI and acne based on patients' beliefs [30]. In a study by Gündüz et al. 72 patients aged 10–18 years were investigated, and it was reported that patients with higher BMI had severe forms of acne [31]. So, it seems that patients with a higher BMI may believe their diet, including fatty and fried food, worsens acne.

We also found that education level was significantly associated with the belief in the impact of diet on acne. Most of the participants in the current study had university education. Few studies evaluated the effect of education on knowledge about acne and dietary effects on acne. Alshammrie et al. conducted an epidemiological study about patients with acne in Saudi Arabia, and their results were similar to ours [32]. Patients with acne who have a higher education level can search easily in different databases about acne‐related factors, and it may increase their perception about the relationship between acne and diet.

Based on our knowledge, previous studies did not assess the relationship between skin type, foods, and acne. We assessed this relationship, and it was found that skin type had a significant relationship with the belief of patients about the effects of diet on acne. Further studies should evaluate this relationship because related data are lacking.

## 5 Conclusion

A remarkable percentage of acne patients believe that diet affects acne. Sweets and chocolates are commonly believed to exacerbate acne, whereas fruits and vegetables are considered beneficial for improving acne. Patients' beliefs about the effects of diet on acne were significantly associated with the severity of acne and skin type. BMI and education level impact patients' perception about the relationship between diet and acne in terms of some foods like fatty and fried foods. It seems that all patients with acne need to be educated about dietary habits that impact acne.

## Author Contributions

F.G., M.S., S.D., Z.T., and Z.R. performed the research. F.G. designed the research study. F.G., M.S., and S.D. contributed essential reagents or tools. Z.R. analyzed the data. F.G., M.S., S.D., Z.T., and Z.R. wrote the paper.

## Conflicts of Interest

The authors declare no conflicts of interest.

## Supporting information


**Data S1.** Data collection instrument.

## Data Availability

The data that support the findings of this study are available from the corresponding author upon reasonable request.
