# Supplementary material for: Evaluating the Association Between Acne Vulgaris and Diet: An Exploratory Study on Patient Beliefs and Perceptions
Source: J Cosmet Dermatol. 2025 Jul 8;24(7):e70285. doi: 10.1111/jocd.70285 (PMC12235578; doi:10.1111/jocd.70285)
Supplement: Supplementary file 1 — Data S1. Data collection instrument. [file JOCD-24-e70285-s001.docx]

# Data Collection Instrument

## Demographic Information

| Question | Options |
| --- | --- |
| Gender | □ Male □ Female |
| Age | □ 12-19 □ 20-29 □ 30-39 □ 40-45 |
| Body Mass Index (BMI) | □ < 18.5 (Underweight) □ 18.5-24.9 (Normal) □ 25-29.9 (Overweight) □ >= 30 (Obese) |
| Education | □ < High School □ High School □ University |
| Occupation | ____________________________________ |
| Height | ____________________________________ |
| Weight | ____________________________________ |

## Skin Characteristics

| Question | Options |
| --- | --- |
| History of Acne | □ Previously □ Currently □ Both |
| Acne Severity | □ Severe □ Moderate □ Mild |
| Skin Type | □ Normal □ Dry □ Oily □ Combination |
| Family History | □ Yes □ No |

## Impact of Diet on Acne

| Question | Options |
| --- | --- |
| Can dietary habits affect acne? | □ Not at all □ Slightly □ Sometimes □ very much □ absolutely |
| Does changing dietary habits improve acne? | □ Yes □ No |

## Various Foods Affecting Acne

| Question | Options |
| --- | --- |
| Spices | □ Aggravates □ Improves □ No Effect □ No Opinion |
| Sweets and Chocolates | □ Aggravates □ Improves □ No Effect □ No Opinion |
| Snacks (Chips and Puffs) | □ Aggravates □ Improves □ No Effect □ No Opinion |
| Nuts (Walnuts, Almonds, Hazelnuts, Pistachios) | □ Aggravates □ Improves □ No Effect □ No Opinion |
| Sunflower Seeds | □ Aggravates □ Improves □ No Effect □ No Opinion |
| Non-Alcoholic Carbonated Drinks | □ Aggravates □ Improves □ No Effect □ No Opinion |
| Milk | □ Aggravates □ Improves □ No Effect □ No Opinion |
| Caffeine (Coffee, Tea) | □ Aggravates □ Improves □ No Effect □ No Opinion |
| Spicy Foods | □ Aggravates □ Improves □ No Effect □ No Opinion |
| Fatty and Fried Foods | □ Aggravates □ Improves □ No Effect □ No Opinion |
| Eggs | □ Aggravates □ Improves □ No Effect □ No Opinion |
| Butter and Cream | □ Aggravates □ Improves □ No Effect □ No Opinion |
| Fruits and Vegetables | □ Aggravates □ Improves □ No Effect □ No Opinion |
| Salty Foods | □ Aggravates □ Improves □ No Effect □ No Opinion |
| Vinegar and Lemon Juice | □ Aggravates □ Improves □ No Effect □ No Opinion |
| Vitamin A | □ Aggravates □ Improves □ No Effect □ No Opinion |
| Vitamin B | □ Aggravates □ Improves □ No Effect □ No Opinion |
| Antioxidants (Vitamin E, Selenium, Green Tea) | □ Aggravates □ Improves □ No Effect □ No Opinion |
| Fish and Omega-3 | □ Aggravates □ Improves □ No Effect □ No Opinion |
| Zinc | □ Aggravates □ Improves □ No Effect □ No Opinion |
